# An overview of the nutritional value, health properties, and future challenges of Chinese bayberry

**DOI:** 10.7717/peerj.13070

**Published:** 2022-03-04

**Authors:** Shuwen Zhang, Zheping Yu, Li Sun, Haiying Ren, Xiliang Zheng, Senmiao Liang, Xingjiang Qi

**Affiliations:** Zhejiang Academy of Agricultural Sciences, Institute of Horticulture, Hangzhou, Jianggan, China

**Keywords:** Chinese bayberry, Botanical characteristics, Bioactive compounds, Nutritional value, Health function

## Abstract

Chinese bayberry (CB) is among the most popular and valuable fruits in China owing to its attractive color and unique sweet/sour taste. Recent studies have highlighted the nutritional value and health-related benefits of CB. CB has special biological characteristics of evergreen, special aroma, dioecious, nodulation, nitrogen fixation. Moreover, the fruits, leaves, and bark of CB plants harbor a number of bioactive compounds including proanthocyanidins, flavonoids, vitamin C, phenolic acids, and anthocyanins that have been linked to the anti-cancer, anti-oxidant, anti-inflammatory, anti-obesity, anti-diabetic, and neuroprotective properties and to the treatment of cardiovascular and cerebrovascular diseases. The CB fruits have been used to produce a range of products: beverages, foods, and washing supplies. Future CB-related product development is thus expected to further leverage the health-promoting potential of this valuable ecological resource. The present review provides an overview of the botanical characteristics, processing, nutritional value, health-related properties, and applications of CB in order to provide a foundation for further research and development.

## Introduction

**Figure 1:**
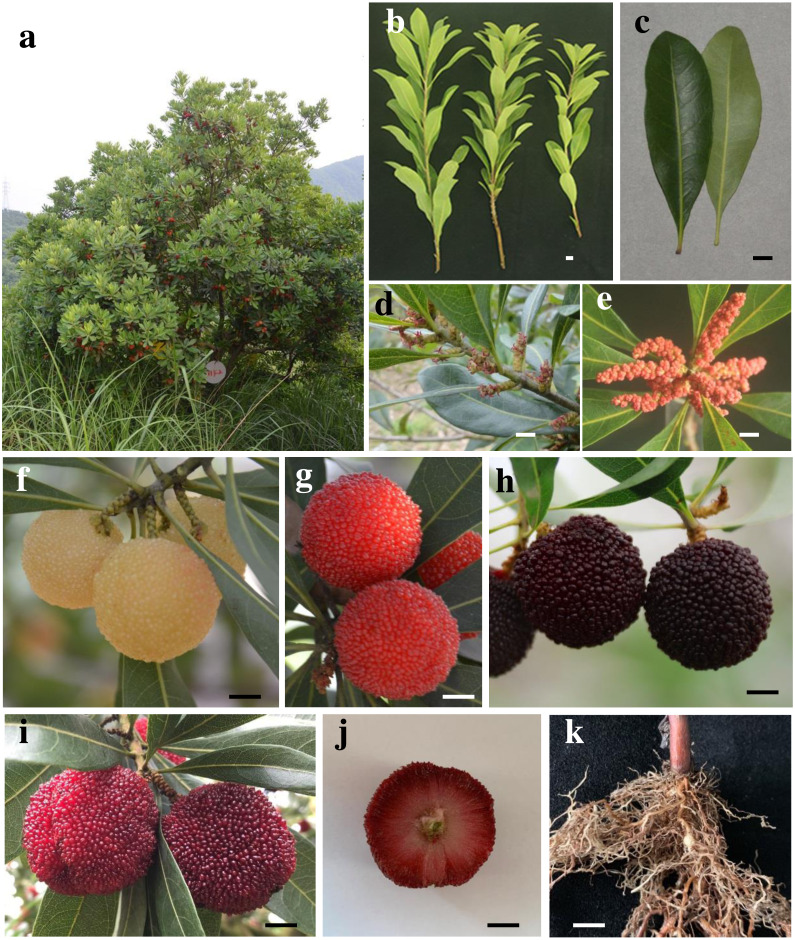
Chinese Bayberry plants and fruits. (A) Tree shape. (B) Twig morphology. (C) Leaf morphology. (D) Female flower. (E) Male flower. (F) White type fruit. (G) Pink type fruit. (H) Black type fruit. (I) Red type fruit. (J) Fruit transverse section. (K) Seedling root system and nodular structure.

Plants in the *Myrica* L. family include *Myrica rubra*, *Myrica esculenta*, *Myrica Nana*, *Myrica Adenophora*, *Myrica cerifera*, *Myrica Faya,* *Myrica Rivas martinezii,* and ∼47 other species (Jia et al., 2015), which are found through Australia, North America, and Southeast Asia (Erickson & Hamrick, 2003). *Myrica* L. family plant roots often exhibit symbiotic relationships with nitrogen fixing rhizobia, including shrubs or small evergreen or deciduous. *Myrica rubra*, which is known as the Chinese Bayberry (CB) and is an important subtropical fruit species in southern China, is the only member of this family that is subject to economic cultivation. It is an evergreen tree with a pleasing shape (Fig. 1A, this tree (∼15 years) that about 2.5 m high was photographed in Hangzhou), and can be planted for fire prevention and for greening purposes. Archaeological investigations of the Hemudu site from the Neolithic Age have revealed that CB plants have been planted for at least 7,000 years. These perennial shrubs are 2–3 m in height on average (maximum: 6 m), with waxy or shiny single alternating leaves that are elliptic or oblanceolate in shape and 8–13 cm in length, with new leaves appearing emerald green with yellow gland spots on the back (Figs. 1B and 1C, the branches and leaves were photographed in Jinhua). CB is a dioecious plant associated with the ZW sex-determination system and catkin inflorescence (Jia et al., 2019; Wang et al., 2020a). The female inflorescence is a long oval ∼1.5 cm in length often found in solitary leaf axils (Fig. 1D, female inflorescence was photographed in Jinhua), whereas the male inflorescence is cylindrical, ∼3 cm in length, and clustered in leaf axils, with green, yellow, red, and other colorations (Fig. 1E, male inflorescence was taken from Jinhua and photographed in the laboratory in Hangzhou). CB fruits are drupes encapsulating a single seed that ripen from mid-May to early July (Sun et al., 2013a), and exhibit a distinctive aroma (Kang et al., 2012). The fruits can be separated into four groups based upon coloration, including white, pink, red, and black type fruits (Figs. 1F and 1I, the mature fruit were photographed in Shaoxing, Ningbo, Taizhou and Jinhua, respectively). The outside of the seed exhibits many dense saccular bodies with a diameter of 2.5−3.6 cm (Fig. 1J), and there is no pericarp covering the surface of the fruit. Representative fruit colors include the Shuijingzhong, Xiazhihong, Dongkui, and Biqizhong varieties, with black varieties being most common. At present, the main varieties are Dongkui and Biqizhong. Of these, Dongkui is a large fruit variety desirable to consumers and producers, while Biqizhong fruits exhibit robust adaptability and a wide planting range and distribution. Both of these varieties are primarily distributed in more than 10 provinces south of the Yangtze River, including Zhejiang, Fujian, Jiangsu, Guangdong, and Hunan. According to the analysis of the National Bureau of statistics in 2020, there are 87,995 hectares devoted to CB cultivation in Zhejiang Province with an output of 654,997 tons valued at 4,699.72 million yuan (RMB) (approximately 740 million USD), underscoring the economic value of this popular fruit.

CB is a nodular plant (Fig. 1K), with annual seedlings exhibiting a height of ∼30 cm, and CB roots fix atmospheric nitrogen to facilitate plant growth through a symbiotic relationship with *Frankia* (Nouioui et al., 2017). *Frankia* strains isolated from CB root nodules exhibit rich genetic diversity (He et al., 2004a; He et al., 2004b). CB plants are highly adaptable and can be readily grown in harsh or barren environments including weakly acidic soil with a minimum tolerated temperature of −9 °C. Notably, CB exhibits a number of ecological benefits, facilitating water and soil conservation, adjusting the microenvironmental climate, controlling the flow of water and associated soil erosion, and thereby reducing the risk of disastrous flooding.

CB is among the most popular and valuable fruits in China owing to its attractive color, unique sweet/sour taste, and medicinal value. In the *Ben Cao Gang Mu* (compendium of materia medica) written by Li Shizhen during the Ming Dynasty, CB is purported to quench thirst, cleanse the stomach and intestines, and harmonize the viscera. Most studies to date have focused on the function of isolated compounds or extracts derived from CB leaves, bark, and fruits, all of which are utilized in traditional Chinese medicine owing to their anti-bacterial, anti-cancer, anti-oxidant, and anti-inflammatory properties. These CB extracts contain high levels of flavonols, phenolic acids, sugars, organic acids, proteins, and vitamins (Xia et al., 2021a; Lyu et al., 2021), with CB fruits containing particularly high levels of flavonoids and phenolic acids, which are regulated by many genes, such as caffeoyl-CoA O-methyltransferase and anthocyanidin 3-O-glucosyltransferase (Ren et al., 2019; Ren et al., 2021).

This review was constructed to provide a comprehensive overview of the nutritional properties, health-related benefits, and applications of CB in an effort to highlight future challenges and trends associated with the use of this economically important plant, in addition to comprehensive characterizing the metabolites within CB fruits in order to provide a foundation for further research and development.

## Survey Methodology

In this review article, we examine the National Center for Biotechnology Information (NCBI), China National Knowledge Infrastructure (CNKI), Web of Science, and Google Scholar platform. In this respect, we searched the keywords to retrieve relevant literature: “Chinese Bayberry or Red Bayberry”, “Chinese bayberry nutrition”, “Chinese bayberry health properties”, “Chinese bayberry *Frankia*” . While current publications between 2002 and 2021 were considered, publications that did not fall within this time period but contained critical information and were relevant to the review’s objectives were also considered. It primarily focuses on the analysis of data published from 2013 to 2021. The *Myrica rubra* database (http://www.bayberrybase.cn/) were used to search relevant researchs and key genes controlling substance synthesis. Since there were no reports on all detectable secondary metabolites in CB fruit in the available references, we use some original data for description. Additionally, the reference lists of the retrieved literature were combed for additional pertinent publications.

## The Nutritional Properties of Chinese Bayberry

### Organic acids and sugars

CB fruits exhibit a please sweet and sour taste. Prior reports have indicated that these fruits contain soluble solid levels ranging from 8.4–15.0% (Cheng et al., 2016; Liang et al., 2019; Liang et al., 2020; Liu et al., 2020; Zhang et al., 2016a; Zhang et al., 2018a; Zhang et al., 2021; Yan et al., 2016), with total sugar and total acid contents of 8.4% and 1.2%, respectively. While the data herein were from multiple sources and were derived from different analytical approaches, we have converted these data into the same units to reduce variability and permit statistical analyses. Sucrose, fructose, and glucose are the main sugars in CB fruits fruit, with respective content levels of 3.8–15.9 g/100 g, 0.9−1.9 g/100 g, and 0.9−1.6 g/100 g, respectively. Organic acids in these fruits primarily include citric acid, malic acid, oxalic acid, tartaric acid, and vitamin C (ascorbic acid), with respective content levels of 7.2–14.0 g/kg, 0.8 g/kg, 25.3 mg/kg, 438.5 mg/kg (Li et al., 2017), and 11.9–114.6 mg/100g (Table 1, values measured on a fresh weight basis), with vitamin C levels being similar to those in strawberries (25.08–108.1 mg/100 g) (Kim et al., 2015; Urün et al., 2021) and citrus fruits (110 mg/100 g) (Elkhatim, Elagib & Hassan, 2018; Rey, Zacarías & Rodrigo, 2020).

**Table 1:** The primary attributes of Chinese Bayberry fruits.

Attributes	Assay method	Range	Average	Reference
Soluble solids (%)	Colorimetric assay	8.4–15.0	11.3	Liang et al. (2019); Liang et al. (2020); Zhang et al. (2018a)
Total sugar (%)	Colorimetric assay	5.8–10.4	8.4	Cheng et al. (2016); Liang et al. (2020); Zhang et al. (2018a)
Glucose (g/100 g)	Chromatography	0.9–1.6	1.2	Cheng et al. (2016); Liang et al. (2019); Zhang et al. (2018a)
Fructose (g/100 g)	Chromatography	0.9–1.9	1.3	Cheng et al. (2016); Liang et al. (2019); Liang et al. (2020);
Sucrose (g/100 g)	Chromatography	3.8–15.9	7.4	Cheng et al. (2016); Li et al. (2017); Zhang et al. (2018a)
Total acid (%)	Colorimetric assay	0.7–2.6	1.2	Cheng et al. (2016); Liang et al. (2019); Liang et al. (2020); Li et al. (2017)
Malic acid (g/kg)	Chromatography	0.3–1.3	0.8	Liang et al. (2019); Liang et al. (2020); Li et al. (2017)
Oxalic acid (mg/kg)	Chromatography	13.3–60.0	25.3	Liang et al. (2019); Liang et al. (2020); Zhang et al. (2018a)
Citric acid (g/kg)	Chromatography	7.2–14.0	11.0	Liang et al. (2019); Liang et al. (2020); Zhang et al. (2018a); Li et al. (2017)
Tartaric acid (mg/kg)	Chromatography	220.6–583.6	438.5	Li et al. (2017)
Vitamin C (mg/100 g)	Colorimetric assay	11.9–114.6	39.7	Li et al. (2017); Liang et al. (2019); Liang et al. (2020); Zhang et al. (2018a)
Total polyphenol (mg/100 g)	Chromatography	61.6–498.9	272.4	Sun et al. (2013a); Xia et al. (2021a);
Total flavonoids (mg/100 g)	Chromatography	13.6–294.3	149.3	Sun et al. (2013a); Xia et al. (2021a); Zhang et al. (2018a)

### Flavonoids

CB fruits contain high levels of flavonoids (13.6–294.3 mg/100 g fresh weight [FW]) (Sun et al., 2013a; Xia et al., 2021a; Zhang et al., 2018a). Flavonoids are key bioactive CB derivatives, with the number of detected polyphenols 38 reported by liquid chromatography quadrupole time-of-flight mass spectrometry (LC-Q-TOF-MS), where proanthocyanidins, as well as flavonols, including myricitrin and quercetrin, were the predominant ingredients in a previous study (Liu et al., 2020). CB-derived flavonoids are thought to exhibit beneficial anti-cancer and anti-diabetes activities (Zhang et al., 2018b). The most abundant flavonoid in these analyzed fruits was the cyanidin-3-O-glucoside (6,322–1,1846 mg/kg dry weight [DW]), followed by the epicatechin (82.25–111.87 mg/kg DW), quercetin (5.98–36.47 mg/kg DW), myricetin-3-O-rhamnoside (21.2–91.6 mg/kg DW), kaempferol-3-O-rhamnoside (279.14 mg/100 g DW), quercetin-3-O-rutinoside (0.07−1.39 mg/kg DW), quercetin-3-O-galactoside (30.8 mg/g DW), quercetin-3-O-glucoside (8.2 mg/g DW) (Liu et al., 2020; Zhang et al., 2016a; Zhang et al., 2021). The total relative anthocyanin levels in these fruits are 2.80−5.12 mg/kg (DW) (Zhang et al., 2021). These substances account for the majority of secondary metabolites present in CB fruits, offering a valuable resource for future studies of the association between flavonoids and physiological function.

### Phenolic acids

Total phenolic content levels in CB fruits range from 61.6–498.94 mg/100 g FW (Sun et al., 2013a; Xia et al., 2021a). These polyphenols exhibit anti-oxidant and anti-proliferative activities, as well as bacteriostatic properties which have led to interest in their use as natural preservatives (Tao et al., 2020). In prior analyses of secondary metabolites, 15 CB-derived phenolic acids were identified (Liu et al., 2020). The content of protocatechuic acid, caffeic acid, and p-coumaric acid levels in CB fruits are 32.12–133.84 mg/kg (DW), 0.39−4.45 mg/kg (DW), and 0.05−0.57 mg/kg (DW) (Zhang et al., 2021), and these compounds have been ascribed beneficial pharmacological activities including the ability to prevent platelet aggregation, reduce myocardial oxygen consumption, increase myocardial oxygen tolerance, and slow the heart rate. In addition, they exhibit bacteriostatic, neuroprotective, analgesic, anti-tumor, and anti-oxidant activity (Song et al., 2020).

CB fruits also contain a variety of other compounds including gallic acid and ellagic acid. Total levels of gallic acid and ellagic acid are 19.81–102.30 mg/kg (DW) and 2.58–10.06 mg/kg (DW), respectively (Zhang et al., 2016a). Studies have shown that these compounds may be of value in the context of treating liver disease, and for preventing retinal diseases associated with oxidative damage, arteriosclerosis, and cerebrovascular diseases (Fu et al., 2014; Liang et al., 2021; Mizuno et al., 2017).

In this review, we summarized 38 flavonoids and 15 phenolic acids that can be detected in CB fruit, and analyzed the content of some of them, which provides a reference for the separation, purification and functional research of secondary metabolites in future research.

## The Health-Related Properties of Chinese Bayberry

### Anti-cancer properties

Some researchers have demonstrated that CB extracts exhibit anti-cancer activity (Wang et al., 2016), with many flavonoids (cyanidin-3-O-glucoside, myricanol, prodelphinidins, proanthocyanidins, and isoquercitrin) having been shown to inhibit apoptosis in a variety of tumor cells (Zhang et al., 2018b).

Many studies have explored the mechanistic basis for the anti-tumor activity of CB extracts. For example, CB fruit-derived cyanidin-3-O-glucoside was shown to suppress gastric adenocarcinoma xenograft growth in a dose-dependent fashion in mice (Wang et al., 2016). Myricanol extracted from CB bark can similarly suppress A549 lung cancer cell growth in a dose-dependent fashion (Dai et al., 2014), while myricitrin (myricetin-3-O-rhamnoside), quercitrin (quercetin-3-rhamnoside), and proanthocyanidins derived from CB leaves inhibited the growth of A2780/CP70 ovarian cancer cells (Zhang et al., 2018b; Zhang et al., 2018c). CB leaf-derived prodelphinidin and proanthocyanidins can further suppress the growth of OVCAR-3 human ovarian cancer cells (Fu et al., 2017; Zhang et al., 2018d). Isoquercitrin extracted from CB fruits was also able to inhibit the viability and colony formation activity of the HepG2 and Huh7 human liver cancer cell lines, activating apoptosis and autophagy dysregulation in these cells (Shui et al., 2020) (Table 2). CB extracts may thus be a valuable resource for natural compounds with anti-tumor activity.

**Table 2:** A summary of studies evaluating the major health-related benefits associated with CB.

Health-promoting benefit	Extract fractions	*In vitro* or *in vivo*	Compound	Tumor cell type or model animal	Research results	Reference
Anti-cancer	Fruit	*In vivo* (mice)	Cyanidin-3-O-glucoside	Balb/c nude mice	Cyanidin-3-glucoside significantly suppressed the growth of SGC-7901 tumor xenografts.	Wang et al. (2016)
	Bark	*In vitro*	Myricanol	A549 human lung adenocarcinoma cells	Myricanol exhibited growth-inhibiting and apoptosis-inducing activities in A549 cells.	Dai et al. (2014)
	Leaves	*In vitro*	Myricitrin, quercetrin, Proanthocyanidins	A2780/CP70 ovarian cancer cells	Flavonoids induced apoptosis and G1 cell cycle arrest in ovarian cancer cells.	Zhang et al. (2018b); Zhang et al. (2018c)
	Leaves	*In vitro*	Prodelphinidins, Proanthocyanidins	OVCAR-3 human ovarian cancer cells	Prodelphinidins and Proanthocyanidins induced apoptosis in OVCAR-3 human ovarian cancer cells.	Fu et al. (2017); Zhang et al. (2018d)
	Fruit	*In vitro*	Isoquercitrin	HepG2 and Huh7 hepatocellular carcinoma cells	Isoquercitrin induced apoptosis and autophagy in hepatocellular carcinoma cells.	Shui et al. (2020)
Anti-oxidant	Leaves, fruit	*In vitro*	Myricitrin, Quercetin-3-O-rhamnoside, Phenolic acids, Anthocyanin	–	Flavonoids and phenolic acids exhibited strong chemical and cellular antioxidant activity.	Zhang et al. (2016b)
	Leaves	*In vitro*	Proanthocyanidins	–	Proanthocyanidins in Chinese bayberry leaves exhibited antioxidant potency.	Fu et al. (2014)
	Fruit	*In vivo* (pigs)	Cyanidin-3-O-glucoside	Three-day-old Duroc/Landrace Large White F1 cross-neonatal pigs	Cyanidin-3-O-glucoside exhibited protective efficacy on neonatal porcine islets.	Li et al. (2017a)
Anti-diabetic	Fruit	*In vivo* (mice)	Proanthocyanidins, Flavonols	KK-A^y^ mice	Fruit extracts significantly reduced fasting blood glucose, elevated glucose tolerance, and insulin sensitivity in diabetic KK-A^y^ mice.	Liu et al. (2020); Zhang et al. (2016a)
	Fruit	*In vivo* (mice)	Cyanidin-3-O-glucoside	Six to 8-week-old immune-deficient C57BL/6-rag1^tm1/mom^ male mice	Cyanidin-3-O-glucoside exhibited protective efficacy on neonatal porcine islets	Li et al. (2017a)
	Fruit	*In vivo* (mice)	Cyanidin-3-O-Glucoside	Pancreatic *β* cells, diabetic mice	Cyanidin-3-glucoside exhibited protective and hypoglycemic effects in diabetic mice.	Sun et al. (2012)
	Fruit	*In vitro*	Flavonoids	–	Flavonoids have the ability to *α*-Glucosidase inhibitory activities.	Yan et al. (2016)
	Leaves	*In vitro*	Proanthocyanidins	–	Proanthocyanidins exhibited *in vitro* inhibitory activity against pancreatic *α*-amylase.	Wang et al. (2020b)
Anti-obesity	Bark	*In vivo* (zebrafish)	Myricanol	High-fat diet-fed zebrafish	Myricanol mitigated lipid accumulation high fat diet-fed zebrafish.	Shen et al. (2019a)
	Leaves	*In vivo* (mice)	Proanthocyanidin	High-fat diet-induced obese rats	Procyanidins exhibited anti-obesity activity in a high-fat diet-induced obese rat model.	Zhou, Chen & Ye (2017)
Neuroprotection	Bark	*In vitro*	Myricitrin, Myricanol	PC12 cells	Myricitrin and myricano l 11-sulfate were shown to be neuroprotective.	Shen et al. (2019b)
Anti-aging	Fruit	*In vitro*	Phenolic extracts	BSA-fructose model	Phenolics inhibited protein glycation and the formation of advanced glycation end-products, and exhibited anti-aging properties.	Zhang et al. (2021)
Anti-inflammatory	Fruit	*In vitro*	Flavonols, Myricitrin, Myricetin	human SZ95 sebocytes	Extracts had effects on anti-inflammatory effects in P. acnes-stimulated human SZ95 sebocytes.	Chen et al. (2019)
Treating cerebral and cardiovascular diseases	Root bark	*In vivo* (mice)	Myricitrin	ApoE −/ −mouse	Myricitrin protects against oxidative stress-induced vascular endothelial cell damage and inhibits early atherosclerosis plaque formation.	Sun et al. (2013b)
	Bark	*In vivo* (mice)	Myricitrin	Male Sprague-Dawley rats	Myricitrin-induced suppression of myocardial apoptosis.	Sun et al. (2016)
	Bark	*In vivo* (mice)	Flavonoids	8 week-old male Sprague-Dawley rats	Flavonoids protected against cardiomyocyte injury.	Wang et al. (2019)
	Fruit	*In vivo* (mice)	Anthocyanins	Male ICR mice	Anthocyanin protected against cerebral ischemia-reperfusion injury.	Cui et al. (2018)

### Anti-oxidant, anti-inflammatory, and anti-aging properties

CB extracts exhibit potent anti-oxidant and free radical scavenging activity within treated cells (Xia et al., 2021b). Both myricitrin and quercetin-3-rhamnoside extracted from CB leaves, as well as anthocyanin extracted from CB fruits, function as potent anti-oxidants (Zhang et al., 2016b; Huang et al., 2014). Similarly, proanthocyanidins derived from CB leaves exhibit pronounced anti-oxidant potency (Fu et al., 2014), while cyanidin-3-O-glucoside extracted from CB fruit can protect neonatal porcine islets against reactive oxygen species-induced injury (Li et al., 2017a). Cyanidin-3-O-glucoside and myricetin additionally exhibit anti-inflammatory activity when used to treat *P. acnes*-stimulated human SZ95 sebocytes, making them promising modulators of inflammatory signaling pathways in the treatment of skin acne (Chen et al., 2019). Phenolic-rich extracts derived from CB can additionally prevent protein glycation and advanced glycation end-products formation, in addition to exhibiting anti-aging properties (Zhang et al., 2021) (Table 2). CB fruits and leaves are an important source of therapeutically useful flavonoids and polyphenolics. These extracts possess strong anti-oxidant anti-inflammatory, and anti-aging properties, and may thus be developed as natural anti-oxidants to benefit public health.

### Anti-diabetic and anti-obesity properties

A number of natural products that exhibit hypoglycemic activity have been identified in recent years (Dong et al., 2021; Hung et al., 2012; Chukwuma et al., 2019), and these compounds are reported to be safe and to additionally possess promising anti-oxidant, anti-tumor, and lipid-lowering activities. As such, they hold promise as alternative therapeutic tools with the potential to alleviate pharmacological dependence upon hypoglycemic and lipid-lowering drugs (Boath, Stewart & McDougall, 2012). Extracts from different CB tissues have been found to exhibit these properties. For example, CB fruit extracts contain high levels of flavonols and proanthocyanidins, and were found to significantly lower fasting blood glucose while increasing glucose tolerance and insulin sensitivity in diabetic KK-A^y^ mice (Liu et al., 2020; Zhang et al., 2016a). Moreover, the expression of the insulin 1 and glycogen synthase kinase 3 *β* genes were notably suppressed whereas hepatic AMPK *α* phosphorylation was significantly increased in treated mice, suggesting that these CB fruit extracts can exert anti-diabetic efficacy at least in part via an AMPK-dependent pathway (Wang et al., 2020b). Cyanidin-3-glucoside-rich fruit extracts can further protect pancreatic *β* cells against oxidative stress-induced injury and associated hypoglycemic effects in diabetic mice (Sun et al., 2012). CB fruits are an excellent natural anti-diabetic food, suggesting their potential application as a functional food ingredient or for further drug discovery use in the prevention and control of diabetes mellitus and its complications.

Myricanol extracts derived from CB bark have been shown to suppress lipid accumulation in zebrafish fed a high-fat diet by inhibiting peroxisome proliferator-activated receptor *γ*, CCAAT/enhancer-binding protein *α*, and other adipogenic factors (Shen et al., 2019a). Moreover, proanthocyanidin extracts derived from CB leaves can exhibit anti-obesity activity owing to the upregulation of SIRT1 and the consequent deacetylation of PPAR- *γ* together with C/EBP- *α* downregulation and BMP4 upregulation to increase brown fat levels in a high-fat diet-induced rat model of obesity (Zhou, Chen & Ye, 2017). CB extracts exhibit significant anti-obesity efficacy, and may thus offer value as a potential therapeutic agent for the treatment of obesity.

### The neuroprotective properties of CB and its therapeutic use in the treatment of cerebrovascular and cardiovascular diseases

CB extracts have also been shown to exhibit neuroprotective activity and to be effective in the treatment of cerebral and cardiovascular diseases. Anthocyanin-rich CB fruit extracts can protect against cerebral ischemia-reperfusion injury in Male ICR mice (Cui et al., 2018), while flavonoids derived from these fruits exert cardioprotective activity by decreasing the severity of oxidative damage in 8-week-old Sprague-Dawley rats (Wang et al., 2019). Moreover, an *in vivo* analysis of ApoE^−/−^ model mice and Sprague-Dawley rats found that myricitrin was able to significantly attenuate Dox-induced myocardial damage, protecting against vascular endothelial cell damage and inhibiting the formation of early atherosclerotic plaques (Sun et al., 2013b; Sun et al., 2016). Myricitrin was also found to be neuroprotective (Shen et al., 2019b). The development of myricitrin and anthocyanin-based flavonoids as novel drugs for treating cerebrovascular and cardiovascular diseases is thus an important area for future research, and CB extracts are a valuable source of such compounds and a food supplement with natural neuroprotective properties.

CB trees grow rapidly and need to be trimmed 2–3 times a year, resulting in a large number of discarded leaves and branches. In traditional production contexts, these leaves and branches will be crushed into slag and serve as raw materials for cheap organic fertilizer. However, in light of this review of the various functions of CB extracts, it is clear that valuable compounds can be extracted from non-fruit tissues including leaves, bark, and roots, highlighting novel production opportunities for these discarded leaves and branches. In the future, these safe to eat, these extracts can be applied for dietary use for the prevention of tumor growth and the enhancement of immunity, offering further economic benefits and extending the value and applications of CB.

## Chinese Bayberry Processing and Commercial Applications

### Harvesting, preservation and storage

CB fruits are susceptible to mechanical damage and water loss, physiological deterioration, and microbial decay, making them poorly suited to storage and transportation , such that they have a post-harvest life of just 1–2 days under ambient temperatures, resulting in severe post-harvest losses (Arbol et al., 2016; Wang et al., 2009). As such, high-quality fresh fruits are most often picked by hand, while processed fruits are gathered using auxiliary harvesting tools. Experienced pickers can harvest intact CB fruits at rates of 8–10 kg per hour.

The storage, preservation, and transportation of these fruits are thus essential to their commercial dissemination. Current approaches to the storage of these fruits include chemical preservation, hot air treatment, cold storage, and controlled atmosphere storage. Controlled atmosphere storage primarily consists of the filling of closed packaging containers with ∼15% CO_2_ or NO_2_ to inhibit ethylene release and fruit respiration, followed by storage at 5−8 ° C, allowing fruits to be preserved for approximately 20 days. Under traditional cold storage (5−8 °C), fruits can generally be preserved for 7 days. Chemical preservation can be achieved by treating fruits with preservatives non-thermal plasma-activated water, methyl jasmonate, and 1-MPC, allowing for preservation for up to 13 d (Wang et al., 2009; Ma et al., 2016). CB fruits can also be treated with hot air (48 °C) for 3 h, followed by storage at 4 °C for about 15 days (Wang et al., 2010; Dai et al., 2021).

### Chinese bayberry juice processing

CB fruits consist of 90–95% flesh and just 5–10% core by weight. Following harvesting, CB fruits can be stored at low temperatures (4 °C or −20 ° C), after which fruits are pressed, filtered, and the core and remaining residues are removed (Fang et al., 2006; Fang et al., 2009), resulting in a juice yield of 73.50–84.00%. The total sugar content in prepared CB juice ranges from 2.32−9.46 g/100 mL, while total acid levels range from 0.57−1.36 g/100 mL. total amino acid levels range from 0.057−1.672 g/L, and total phenolic acid and flavonoid contents range from 1.149−2.243 mg/L and 286-907 mg/L, respectively (Tian et al., 2019; Wang et al., 2012; Xu et al., 2014). Polypropylene membrane-filtered CB juice can then be pasteurized, processed, and drunk directly following dilution to taste or stored at −20 °C for up to 180 days.

### Chinese bayberry wine processing

According to prior reports, to prepare CB wine, fresh or frozen CB fruits are crushed using a juice extractor and sucrose is used to adjust the total soluble must content to 15.0° Brix. Musts are then pasteurized for 5 min at 100 °C followed by rapid cooling to 20 °C (Zhang, Li & Fan, 2019). Fermentation is performed using a wild yeast strain isolated from a natural CB mash and identified as *Pichia kluyveri* (Du et al., 2016; Li et al., 2017b), with fermentation being conducted for 4–7 days at 28 °C until the total weight loss is less than 0.2 g/d. After fermentation, wine is clarified for 1 h with 0.20 g/L poly-vinylpyrrolidone and 0.06 g/L chitosan. Following centrifugation, wines are bottled with equal headspace volume and stored for 70 days in the dark at room temperature (Xu et al., 2014). The resultant wine exhibits a total anthocyanin content of 51.1–116.6 mg/L, as well as an ester content of 25.713 mg/L. The primary esters in the resultant wine include ethyl decanoate (9.166 mg/L), ethyl octanoate (6.245 mg/L), ethyl acetate (3.462 mg/L), diethyl butanedioate (2.741 mg/L), and ethyl dodecanoate (2.219 mg/L), respectively. Total acid levels range from 0.323–0.907 mg/L (Cao, Wu & Weng, 2020), while total phenols and flavonoids range from 90.10–510 mg/L following fermentation.

### Culinary and commercial applications

CB fruits exhibit a sugar/acid ratio of approximately 7–15, accounting for their sweet and sour taste. These fruits can promote digestion and control the composition of the gut microbiome (Tao et al., 2020), and have long been used to produce a range of food products and other commercial items. Broadly, these products can be classified into two main categories: beverages, and foods. Beverages include CB juice (Fang et al., 2006), wine (Zhao et al., 2019; Zhang et al., 2020), and sparkling water, while CB fruit-derived foods (Fang, Zhang & Wang, 2007) include dried CB fruits, CB jam, canned CB fruits, candies, ice cream, yam cake, steamed cake, and moon cake.

### CB fruit dry powder

In one proof of concept study, frozen CB juice was thawed and mixed at a 1:1 ratio with a maltodextrin solution (11° Brix), yielding a total solids content in the final solution of 11° Brix. This solution was then fed through a mini spray-dryer (aspirator rate: 100%, 35 m^3^/h; atomization air rotameter: 30 mm, 439 L/h with a co-current flow; drying air inlet temperature: 150 ° C) with the pump rate being adjusted to maintain an 80  °C outlet temperature. When air inlet temperatures were below 50 °C at the end of the experiment, samples were collected (Fang & Bhandari, 2012). These spray-dried protects exhibited stable polyphenol content and anti-oxidant activity over a 6-month storage period (Fang & Bhandari, 2011). Dry powders are the most effective means of preserving the active substances in CB fruits at present, and can also be directly leveraged for further processing into health products or additives.

## Future Challenges and Trends

The fruits, leaves, and bark of CB plants contain many potent bioactive compounds including cyanidin-3-O-glucoside, myricanol, isoquercitrin, myricitrin, isoquercitrin, myricetin, proanthocyanidins, anthocyanins, and flavonols. Several of these compounds exhibit promise for use in industrial applications and warrant further study. For example, proanthocyanidins are sustainable amphiphilic materials with several promising health-promoting properties, thus holding significant promise for use as biomaterials in the context of compound encapsulation. Indeed, proanthocyanidins from CB leaves (Bayberry Leaf Proanthocyanidins, BLPs) can encapsulate oils in the form of spherical microcapsules with controlled morphological characteristics (Pan et al., 2020). Physicochemically stable emulsions have also been developed using a self-assembling colloidal complex composed of BLPs and gelatin (Chen et al., 2020). These microcapsules and emulsifiers offer significant promise for future use in the preparation of food-grade emulsions.

The promising nutritive and health-related benefits of certain secondary metabolites present within CB fruits remain to be assessed. For example, gallic acid has been shown to be effective for the treatment of liquefied petroleum gas poisoning (Akinmoladun et al., 2021), and intestinal parasites (Bouaziz et al., 2021), and in smokers, it can also reverse the negative impacts of nicotine on male fertility (Jalili et al., 2021). As CB fruits contain relatively high gallic acid levels (7.30 × 10^6^), further research into the health-related benefits of this CB-derived compound is warranted. Additionally, peonidin-3-O-glucoside is reported to exhibit anti-depressant activity (Kurnianingsih et al., 2021), to inhibit COVID-19 (Majumder & Mandal, 2020), and to serve as an effective anti-tumor agent (Zhou et al., 2018). Additional research regarding the multipotent activity of peonidin-3-O-glucoside in a range of pathological contexts is thus warranted. Future CB-related product development is thus expected to further leverage the health-promoting potential of this valuable ecological resource.

At present, CB remains an underutilized plant species, primarily owing to difficulties associated with the harvesting, preservation, storage, and transportation of these fruits. As such, new approaches to extending the national and international benefits of CB are needed, including the development of professional and practical harvesting strategies based on fruit and planting environment characteristics, as well as additional research aimed at optimizing efficient storage, preservation, and transportation technologies. Further efforts to market these fruits based upon their exceptional nutritional value and wide applicability in culinary and pharmaceutical contexts are also warranted.

With the development of genomic, transcriptomic, metabolomic, and other high-throughput omics platforms, CB genomic data have become publically available (Jia et al., 2019; Ren et al., 2019), providing a valuable sequence reference for researchers. These omics-based platforms can thus be used for joint analyses aimed at deeply mining genes associated with active functional compounds within CB fruits These platforms additionally provide a basis for in-depth analyses of the regulation of these bioactive compounds, in addition to highlighting opportunities for the *in vitro* synthesis these compounds and their functional validation.

The nitrogen-fixing functions of CB have not been studied in detail to date. In the future, such nitrogen fixation activity warrants further development and may make these plants suitable for growing in barren mountains and evergreen regions. This may enable to the vigorous development of CB into ecological economic forests, thereby maintaining water and soil to improve local environmental conditions.

## Conclusions

In summary, CB exhibits excellent nutritional value owing to the high levels of sugar, vitamin C, anthocyanins, flavonoids, proanthocyanidins, and phenolic acids in these fruits. The primary stages of CB processing include harvesting, fruit preservation, juice pressing and filtering, and wine fermentation. CB exhibits beneficial anti-cancer, anti-oxidant, anti-inflammatory, anti-diabetic, neuroprotective, anti-aging, and anti-obesity properties in addition to offer value in the treatment of cardiovascular and cerebrovascular diseases. CB is currently underutilized in culinary contexts, and has great potential to be incorporated into various foods including high value-added products. CB contains a variety of bioactive compounds with medicinal and therapeutic benefits, and the exploitation of these compounds may offer a valuable resource for new product development.

## Additional Information and Declarations

### Data Availability

The following information was supplied regarding data availability: This is a literature review.
